# Effects of Exercise Training on Renal Carnitine Biosynthesis and Uptake in the High-Fat and High-Sugar-Fed Mouse

**DOI:** 10.3390/molecules25092100

**Published:** 2020-04-30

**Authors:** Aman Upadhyay, Layla Al-Nakkash, Tom L. Broderick

**Affiliations:** 1 Department of Biomedical Sciences, College of Graduate Studies, Midwestern University, Glendale, AZ 85308, USA; aupadhyay19@midwestern.edu; 2 Department of Physiology, College of Graduate Studies, Midwestern University, Glendale, AZ 85308, USA; lalnak@midwestern.edu; 3 Department of Physiology, Laboratory of Diabetes and Exercise Metabolism, College of Graduate Studies, Midwestern University, Glendale, AZ 85308, USA

**Keywords:** exercise, obesogenic, carnitine, γ-butyrobetaine hydroxylase, organic cation transporter

## Abstract

(1) Background: Diet-induced obesity inhibits hepatic carnitine biosynthesis. Herein, the effects of high-fat (HF) and high-sugar (HFHS) feeding and exercise training (ET) on renal carnitine biosynthesis and uptake were determined. (2) Methods: Male C57BL/6J mice were assigned to the following groups: lean control (standard chow), HFHS diet, and HFHS diet with ET. ET consisted of 150 min of treadmill running per week for 12 weeks. Protein levels of γ-butyrobetaine hydroxylase (γ-BBH) and organic cation transporter-2 (OCTN2) were measured as markers of biosynthesis and uptake, respectively. (3) Results: HFHS feeding induced an obese diabetic state with accompanying hypocarnitinemia, reflected by decreased free carnitine levels in plasma and kidney. This hypocarnitinemia was associated with decreased γ-BBH (~30%) and increased OCTN2 levels (~50%). ET failed to improve the obesity and hyperglycemia, but improved insulin levels and prevented the hypocarnitinemia. ET increased protein levels of γ-BBH, whereas levels of OCTN2 were decreased. Peroxisome proliferator-activated receptor-alpha content was not changed by the HFHS diet or ET. (4) Conclusions: Our results indicate that ET prevents the hypocarnitinemia induced by HFHS feeding by increasing carnitine biosynthesis in kidney. Increased expression of OCTN2 with HFHS feeding suggests that renal uptake was stimulated to prevent carnitine loss.

## 1. Introduction

Carnitine is a cofactor that exerts a fundamental role in the oxidation of long-chain fatty acids (FAs). Carnitine and the carnitine shuttle system translocate FAs from the cytosol into the mitochondria where these substrates undergo β-oxidation for the production of energy [1]. Both dietary sources of carnitine and endogenous biosynthesis are critical in regulating carnitine homeostasis. Biosynthesis originates from protein degradation of methionine and lysine, and although the reactions that follow exist in most tissues, the final step in this pathway, which involves the hydroxylation of γ-butyrobetaine (BB) to carnitine by γ- butyrobetaine hydroxylase (γ-BBH), occurs in the liver and kidney [2,3]. Following the release of carnitine into the plasma, the sodium-dependent organic cation transporter-2 (OCTN2) facilitates the uptake of carnitine into peripheral tissues [4]. Expressed in kidney, this transporter has a high affinity for carnitine, and it is important in renal reabsorption of this cofactor from the urine [4]. Further, BB released from tissues enters hepatic and renal cells by the OCTN2 transporter, where it is then converted to carnitine by γ-BBH [5]. The importance of carnitine biosynthesis is supported by the observation that carnitine deficiency syndromes are associated with reduced FA oxidation in tissues causing lipid storage myopathy, muscle weakness, decreased exercise capacity, and cardiomyopathy [6,7].

Studies in rodents have shown that endogenous carnitine biosynthesis and uptake are stimulated with fasting and caloric restriction [8,9]. This has been attributed to an increase in the release of non-esterified FAs (NEFAS) from adipose tissue, resulting in the activation of peroxisome proliferator-activated receptor-alpha (PPARα) [10,11]. PPARα is a ligand-activated transcription factor that stimulates hepatic and renal gene expression of γ-BBH, OCTN2, and carnitine palmitoyltransferase-I (CPT-1) to enhance mitochondrial FA oxidation [8,9,12,13]. We have shown that expression of γ-BBH and OCTN2 is increased in rat and mouse tissue in response to acute and chronic exercise training (ET), with both conditions stimulating carnitine biosynthesis and increasing the levels of plasma carnitine [14,15,16]. 

The effects of diet-induced obesity (DIO) from high-fat (HF) feeding and exercise training (ET) on carnitine homeostasis has been reported. Feeding mice an obesogenic diet by increasing the metabolizable contribution from lipids inhibited protein expression of γ-BBH and OCTN2 in liver, producing a significant reduction in the level of free carnitine (FC) in both liver and plasma [17,18]. ET stimulated the expression of these proteins in liver but failed to improve the concentration of FC in the plasma. The effects of DIO combined with ET on the expression of γ-BBH and OCTN2 in kidney, however, are not fully understood. By stimulating biosynthesis and preventing urinary carnitine loss, the importance of kidney in maintaining carnitine homeostasis is increasingly important during ET, as the demand for carnitine to facilitate FA oxidation in tissues is increased [14,15]. Further, the effects of ET and high fat (HF) feeding supplemented with sugar added in the drinking water, which can potentially further increase insulin resistance compared to a diet consisting of HF alone, remains to be determined. Therefore, in this study, we examined the effects of ET on the expression of γ-BBH and OCTN2 in kidney of mice fed a HF and high-sugar (HFHS) diet over a period of 12 weeks. 

## 2. Results

Body weights were measured at the start and the end of the 12-week feeding and ET study (Table 1). Initial body weights were not different between groups of mice (*p* = 0.4417). As expected, 12 weeks of HFHS feeding resulted in significant changes in final body weights. Mice fed the HFHS diet were obese with significantly higher weights than mice fed a standard diet. In these obese mice, the higher body weight was associated with increased liver and total adipose tissue weights compared to lean mice, whereas no differences in kidney weight were observed (*p* = 0.4828). HFHS feeding also induced hyperinsulinemia and hyperglycemia, although the increase in blood glucose levels in these mice did not reach statistical significance (*p* = 0.0682). The elevated levels of fructosamine following HFHS feeding indicated poor metabolic control. After ET, HFHS-fed mice remained obese, and blood glucose levels did not differ between the non-exercising and exercising mice (*p* = 0.6503). However, insulin and fructosamine levels were improved with ET.

**Figure 1:**
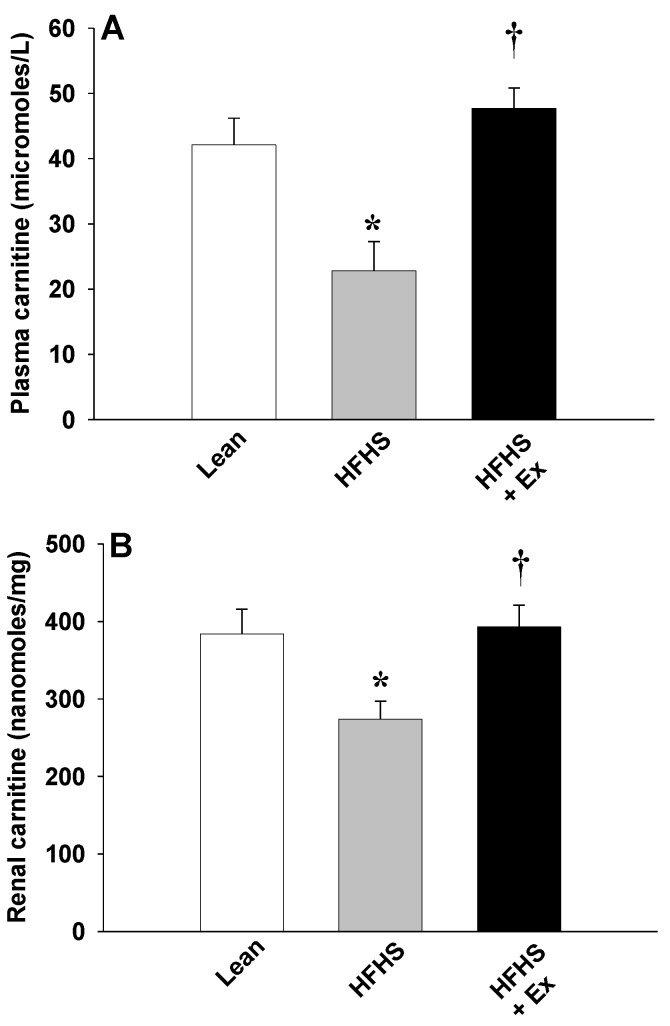
Effects of 12 weeks of HFHS feeding and exercise training on free carnitine levels in plasma (**A**) and kidney (**B**). Values are reported as mean ± SEM for 6 mice in each group. * *P* < 0.05 compared to lean control mice; ^†^ compared to HFHS-fed mice.

**Figure 2:**
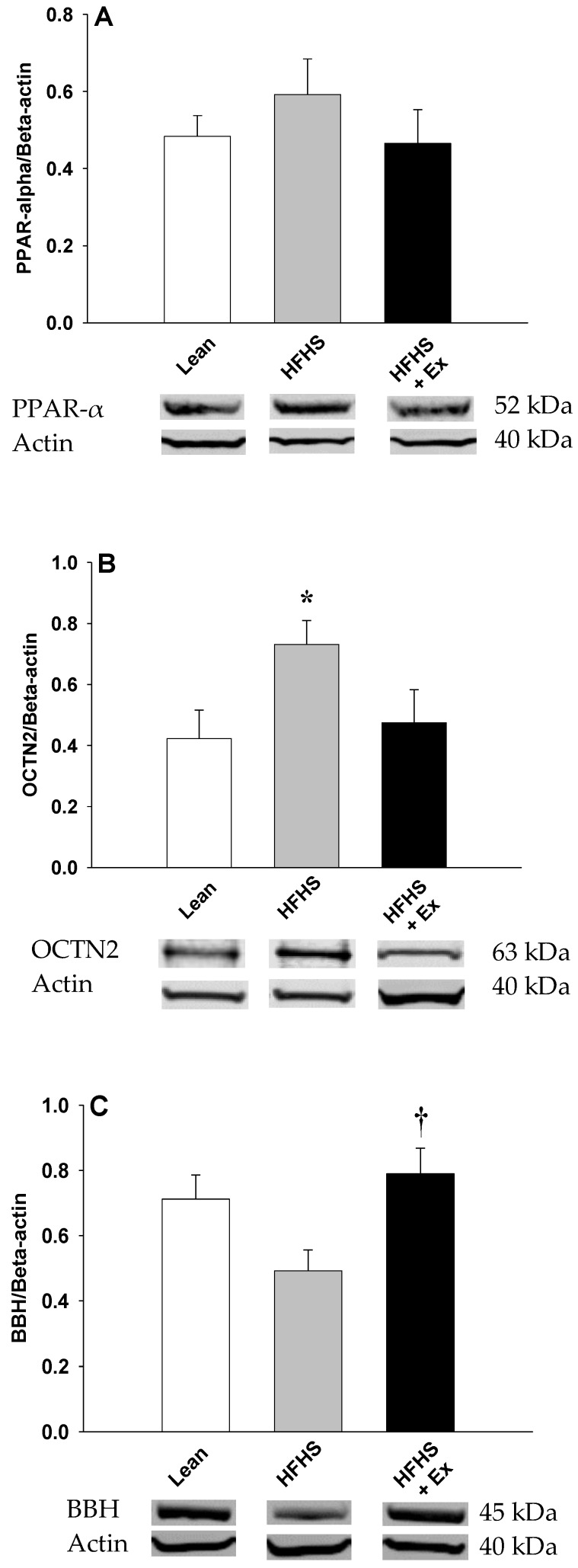
Effects of 12 weeks of HFHS feeding and exercise training on protein expression of PPAR-α (**A**), OCTN2 (**B**), and γ-BBH (**C**) in mouse kidney. Values are reported as mean ± SEM for 4–6 mice in each group. * *P* < 0.05 compared to lean control mice; ^†^ compared to HFHS-fed mice. PPAR-α: peroxisome proliferator-activated receptor alpha, OCTN2: organic cation transporter-2, γ-BBH: γ-butyrobetaine hydroxylase.

The effects of HFHS feeding in mice on plasma FC levels are shown in Figure 1. HFHS feeding resulted in a significant decrease (~50%) in the levels of FC in plasma (Figure 1A) and kidney (Figure 1B). To determine whether this hypocarnitinemia was attributed to decreased biosynthesis, expression of γ-BBH was determined in kidney. As illustrated in Figure 2, HFHS feeding induced a non-significant decrease (~30%, p = 0.0693) in γ-BBH expression (Figure 2C). When OCTN2 was measured to determine the effects on carnitine uptake, protein content was significantly increased, suggesting that renal uptake mechanisms were stimulated (Figure 2B).

Twelve weeks of ET prevented the carnitine deficiency induced by HFHS feeding (Figure 1). Compared to the HFHS-fed mice, this was associated with a significant increase in γ-BBH expression (Figure 2C), resulting in an increase in FC levels in plasma (Figure 1A) and kidney (Figure 1B). OCTN2 levels, on the other hand, were reduced (~37%, *p* = 0.0721) with ET (Figure 2B). Protein levels of PPARα were unchanged (*p* = 0.4988) after 12 weeks of HFHS feeding or ET (Figure 2A). 

## 3. Discussion

Enhanced carnitine biosynthesis and reabsorption from renal routes are adaptations of ET that improve overall carnitine homeostasis. These adaptions occur to meet the increased requirements for FA oxidation by tissues [19]. Hepatic γ-BBH expression is stimulated to increase the carnitine concentrations in plasma, whereas renal OCTN2-mediated uptake of carnitine prevents urinary loss [14,15]. Here, we examined the effects of chronic overnutrition from HFHS feeding and ET on carnitine biosynthesis and uptake in mouse kidney. Our results show that caloric overload induced an obese state with insulin resistance and hypocarnitinemia. This disruption in carnitine homeostasis from obesogenic feeding is consistent with earlier studies, in which mice were fed a HF diet for a similar 12-week period [17,18]. Indeed, chronic overnutrition from HF feeding inhibited mRNA and protein expression of γ-BBH in liver and muscle, leading to low tissue, plasma carnitine, and increased urinary carnitine loss [18]. We extend these observations by demonstrating that HF feeding, with the addition of refined sugars to the drinking water, reduced carnitine biosynthesis in kidney. This state of carnitine deficiency was associated with lower expression of γ-BBH in kidney. Of note was the greater OCTN2 content detected in kidney following HFHS feeding. However, this increase in OCTN2 was not associated with concomitant increases in the lipid-sensing nuclear receptor PPAR-α. Earlier studies have indicated that OCTN2 expression is dependent on the activation by PPAR-α and further confirmed by the observation that PPAR-α null mice develop carnitine deficiency from low OCTN2 expression [12,20]. However, recent evidence indicates that changes in OCTN2 expression in tissues do not always correlate with PPAR-α levels. Ringeis et al. [17] demonstrated that 12 weeks of HF feeding with moderate intensity of ET increased PPAR-α mRNA expression in gastrocnemius muscle without producing a concomitant change in the expression of OCTN2. This is in contrast with the effects observed with acute high intensity exercise, in which increases in PPAR-α protein in kidney, liver, and heart were associated with accompanying increases in OCTN2 expression [16,17]. Similar responses were reported following 10 weeks of twice daily treadmill running in rat kidney and liver [14]. The reasons for these discrepancies in PPAR-α and OCTN2 expression are unclear, but they may relate to the caloric load of the energy-rich diets and exercise intensity. OCTN2 has a high affinity for carnitine in renal filtrate and is considered a key transporter in the reabsorption of carnitine. Thus, it is possible that the increase in OCTN2 expression with HFHS feeding indicated that renal mechanisms were upregulated to prevent carnitine loss and to overcome the reduction in biosynthesis. Twelve weeks of ET were beneficial in preventing the hypocarnitinemia associated with caloric overload. ET stimulated the expression of γ-BBH, resulting in an increase in FC levels in plasma and kidney, indicating increased biosynthesis. This effect of ET in the presence of high caloric intake is consistent with earlier studies in which an increased expression of γ-BBH was reported in other tissues [17]. A key finding in this study was that ET resulted in a downregulation in OCTN2 expression in kidney. The reason why the expression of this transporter is decreased while other studies demonstrate that an increase in the expression in tissues occurs with ET remains to be determined. Nonetheless, our results show that ET induced reciprocal changes in the expression of γ-BBH and OCTN2, suggesting that when biosynthesis is stimulated, the role of renal uptake in preserving FC carnitine levels in plasma is diminished. 

## 4. Material and Methods

### 4.1. Mouse Model and Treatment Protocols

Male C57BL/6J mice aged 4–5 weeks were purchased from Charles River (Wilmington, MA, USA). Mice were housed two per cage in an animal care facility with 12:12-hour light-dark cycle. After a week of acclimatization, mice were randomly divided into three groups (n = 6/group): (1) standard diet with normal water (Lean), (2) high-fat high-sugar (HFHS), and (3) HFHS and exercise (ET). The HF diet contained 60% of energy from lipids, 20% as carbohydrates, and 20% as protein (Dyets Inc., Bethlehem, PA, USA). Mice in the HF groups were also fed sugar in the drinking water (42g/L: 55% fructose/45% sucrose). Mice were provided with food and water ad libitum. The duration of the study was 12 weeks. The Midwestern University Institutional Animal Care and Use Committee (Project MWU-2880, approved 11.20.2018) approved the procedures of this study.

ET consisted of daily treadmill (Exer 3/6, Columbus Instruments, Columbus, OH, USA) running sessions for a total of 150 min per week (30 min/day at 12 meters/min for 5 days) at an intensity corresponding to an estimated oxygen consumption of ~45–48 mL/kg/min based on treadmill belt speed [21]. In addition, this exercise regimen is known to stimulate markers of fatty acid oxidation in tissues of mice with DIO [15]. The protocol was designed to mimic the recommendations for physical activity established by the American College of Sports Medicine and American Diabetes Association [22]. 

### 4.2. Tissue and Plasma Collection

Forty-eight hours after the last exercise, mice were euthanized by asphyxiation in an atmosphere of 100% CO_2_. A surgical thoracotomy inducing pneumothorax was performed and blood was obtained by cardiac puncture. Blood was centrifuged and the plasma separated and assayed for glucose (Wako Diagnostics, Mountain View, CA, USA), insulin (Alpco Diagnostics, Salem, NH, USA), fructosamine, and free carnitine (Abcam, Cambridge, MA, USA) according to manufacturer specifications. Tissues were weighed and the total adipose content was expressed as the sum of visceral, retroperitoneal, and inguinal fat pad weights. Kidneys were snap frozen in liquid nitrogen for protein analysis and measurement of FC levels (Abcam, Cambridge, MA, USA).

### 4.3. Western Blot Analysis

Protein content in renal tissue was measured as previously described [14]. Following homogenization with EDTA, mammalian protease arrest and protein extraction (Tissue PE LB, BD Biosciences, San Jose, CA, USA), samples were analyzed for protein content using a Pierce^TM^ BCA protein assay kit (Thermo Scientific, Rockford, IL). Tissue was then subjected to immunoblot analysis with primary antibodies (PPARα, OCTN2, γ-BBH, 1:1000, Abcam, Cambridge, MA, USA) followed by incubation with the secondary antibody (anti-rabbit immunoglobulin, 1:15,000, Thermo Fisher Scientific, Inc., Waltham, MA, USA). β-actin was used as an internal loading control (1:10,000; Millipore Sigma). Membranes were imaged and band intensity quantified using Odyssey-Clx and Image Studio Software (Li-COR Biosciences, Lincoln, NE, USA). 

### 4.4. Statistical Analysis

Data are reported as the mean ± SEM. Group mean differences were determined using a one-way analysis of variance, followed by a Tukey-Kramer comparison for post hoc analysis. A value of *P* < 0.05 was considered statistically significant.

## 5. Conclusions

Our results indicate that hypocarnitinemia is a consequence of chronic obesogenic feeding in mice. ET reversed this metabolic aberration by enhancing renal carnitine biosynthesis and uptake. This effect of ET occurred independently of the obesity and hyperglycemia in mice. Preventing disrupted carnitine homeostasis through regular ET may benefit patients with metabolic disorders, including carnitine deficiency syndromes and type 1 diabetes. Increasing carnitine biosynthesis by engaging in regular ET can potentially restore FA oxidation in tissues of patients with carnitine deficiency. In addition, for the management of type 1 diabetes, ET may present urinary losses of carnitine by increased OCTN2 mechanisms. 

## Figures and Tables

**Table 1:** The effects of exercise training and HFHS (high-fat high sugar) feeding on physical characteristics of mice.

	Lean	HFHS	HFHS + Exercise
*Body weight (g)*			
Initial body weight	23.5 ± 0.4	22.5 ± 0.9	22.2 ± 0.8
Final body weight	37.6 ± 0.9	47.9 ± 2.3 *	45.7 ± 2.0 *
*Organ weight (g)*			
Liver	1.39 ± 0.08	2.34 ± 0.43 ^‡^	1.89 ± 0.16
Kidney	0.41 ± 0.02	0.40 ± 0.02	0.44 ± 0.03
Total fat	2.21 ± 0.41	4.57 ± 0.33 *	4.33 ± 0.40 *
*Plasma*			
Glucose (mg/dL)	149 ± 12	236 ± 26	216 ± 34
Insulin (ng/mL)	0.46 ± 0.06	1.07 ± 0.09 *	0.62 ± 0.06 ^†^
Fructosamine (μmol/L)	114 ± 14	279 ± 22 *	188 ± 18 *^,†^

Values are reported as mean ± SEM for 6 mice per group. ^‡^ *p* = 0.0537, * *P* < 0.05 compared to lean control mice. ^†^ *P* < 0.05 compared to HFHS-fed mice.
